# High-temperature water–rock interactions and hydrothermal environments in the chondrite-like core of Enceladus

**DOI:** 10.1038/ncomms9604

**Published:** 2015-10-27

**Authors:** Yasuhito Sekine, Takazo Shibuya, Frank Postberg, Hsiang-Wen Hsu, Katsuhiko Suzuki, Yuka Masaki, Tatsu Kuwatani, Megumi Mori, Peng K. Hong, Motoko Yoshizaki, Shogo Tachibana, Sin-iti Sirono

**Affiliations:** 1 Department of Earth and Planetary Science, University of Tokyo, Bunkyo 113-0033, Japan; 2 Laboratory of Ocean-Earth Life Evolution Research, Japan Agency for Marine-Earth Science and Technology, Yokosuka 237-0061, Japan; 3 Research and Development Center for Submarine Resources / Project Team for Next-Generation Technology for Ocean Resources Exploration, Japan Agency for Marine-Earth Science and Technology, Yokosuka 237-0061, Japan; 4 Institut für Geowissenschaften, Universität Heidelberg, Heidelberg 69120, Germany; 5 Institut für Raumfahrtsysteme, Universität Stuttgart, Stuttgart 70569, Germany; 6 Laboratory for Atmospheric and Space Physics, University of Colorado, Boulder, Colorado 80303, USA; 7 Department of Solid Earth Geochemistry, Japan Agency for Marine-Earth Science and Technology, Yokosuka 237-0061, Japan; 8 Department of Natural History Science, Hokkaido University, Sapporo 060-0810, Japan; 9 The University Museum, University of Tokyo, Bunkyo 113-0033, Japan; 10 Department of Earth and Planetary Science, Tokyo Institute of Technology, Meguro 152-8551, Japan; 11 Graduate School of Environmental Science, Nagoya University, Nagoya 464-8601, Japan

## Abstract

It has been suggested that Saturn's moon Enceladus possesses a subsurface ocean. The recent discovery of silica nanoparticles derived from Enceladus shows the presence of ongoing hydrothermal reactions in the interior. Here, we report results from detailed laboratory experiments to constrain the reaction conditions. To sustain the formation of silica nanoparticles, the composition of Enceladus' core needs to be similar to that of carbonaceous chondrites. We show that the presence of hydrothermal reactions would be consistent with NH_3_- and CO_2_-rich plume compositions. We suggest that high reaction temperatures (>50 °C) are required to form silica nanoparticles whether Enceladus' ocean is chemically open or closed to the icy crust. Such high temperatures imply either that Enceladus formed shortly after the formation of the solar system or that the current activity was triggered by a recent heating event. Under the required conditions, hydrogen production would proceed efficiently, which could provide chemical energy for chemoautotrophic life.

## Abstract (web summary)

Observations indicate that the southern hemisphere of Enceladus is geologically active, with spray containing Si nanoparticles being ejected from an underground ocean. Here, the authors report that experiments to constrain reaction conditions suggest the core is similar to that of carbonaceous chondrites.

## Graphical abstract

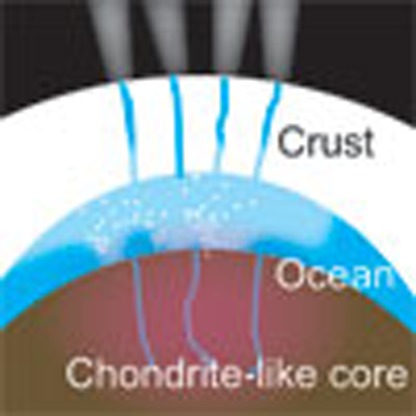

Water-rich plumes of vapour and ice particles with sodium salts erupting from warm fractures near the south pole of Saturn's icy moon Enceladus suggest the presence of a liquid water reservoir in the interior^1^,^2^,^3^,^4^. Recent work combining Cassini measurements and experimental results shows that some of the observed plume materials are associated with ongoing hydrothermal activity in the interor^5^. Nanometre-sized silica particles with a confined size range detected by the Cassini Cosmic Dust Analyser are found to have originated from Enceladus^5^. Supported by the results of hydrothermal experiments, it is indicated that these particles originated from nanosilica colloids that formed when silica saturation was reached upon cooling of hydrothermal fluids^5^. The presence of these particles provides tight constraints on the particular conditions of the interior ocean; that is, the presence of high-temperature reactions (≥∼90 °C), moderate salinity (≤∼4%), and alkaline seawater (pH=8.5–10.5)^5^. Products of ongoing hydrothermal reactions would have been transported upwards from an interior ocean located at a depth of ∼30 km beneath the surface at Enceladus' south pole^6^,^7^ and would have then been ejected into the plume^8^.

One major difference between hydrothermal reactions on Enceladus and those currently occurring on Earth is the plausible presence of abundant primordial volatiles (for example, NH_3_ and CO_2_) provided from icy planetesimals that formed the Saturnian system^9^, and these volatiles are abundant in the gas component of Enceladus' plumes^3^,^10^. However, detailed laboratory investigations on the fate of these volatiles under hydrothermal conditions within Enceladus have not been performed. In addition, Na^+^ is a major constituent in Enceladus' alkaline ocean^1^,^11^,^12^, as Cassini has detected sodium salts, such as NaHCO_3_ and NaCl, in the plume's ice grains^1^,^2^.

Another possible difference between hydrothermal reactions on Enceladus and those currently occurring on Earth is the rock composition. Given the presence of olivine and pyroxene in comets^13^,^14^, these primitive crystalline silicates would also have been two of the most abundant constituents in the building blocks of Enceladus. If the rocks of Enceladus have not experienced large-scale silicate melting throughout its history, the composition of the core would have been chondritic^11^, containing abundance of these primitive minerals. On the other hand, if Enceladus' rocky core has experienced silicate melting in the early stages of its evolution, a more ultramafic, olivine-rich rocks would have been formed within the core, similar to Earth's upper mantle and as proposed for the interior of Ceres^15^. The low-density rocky core suggested by Cassini's data^6^,^7^ (for example, ∼2.4–2.5 g cm^−3^ for an H_2_O mantle with 60 km thickness) is consistent with the presence of hydrous minerals with significant porosity, suggesting the widespread occurrence of water–rock interactions in the core and a supply of aqueous fluid to the subsurface ocean.

To constrain the conditions of hydrothermal reactions on Enceladus, the present study provides the results of further hydrothermal experiments, temporal variations in fluid composition and microscope observations of rock residues collected after the experiments. The experimental results are compared with chemical equilibrium calculations. Based on the results of the experiments, the present study constrains both the reactions of primordial volatiles and the composition of the rock core within Enceladus. Although the previous study^5^ shows that the minimum temperatures of hydrothermal reactions required for the formation of silica nanoparticles depend on a pH change of fluids, it does not discuss the mechanisms or possible range of the change. In the present study, we discuss the range of pH changes and required temperature conditions based on the detailed experiments. Finally, we propose thermal evolution scenarios that could support ongoing hydrothermal activity within Enceladus.

## Results

### Hydrothermal simulations

In the experiments, we used two types of starting minerals with low and high Si contents: a powdered San Carlos olivine (olivine experiment), and a mixture of powdered orthopyroxene (opx) (orthoenstatite: 70 wt.%) and San Carlos olivine (30 wt.%) (opx experiment) (see Methods). The opx experiment (Mg/Si=∼1.2) simulates the alteration of a relatively Si-rich rocky core that has not experienced silicate melting, such as a parent body of carbonaceous chondrites in terms of Mg/Si ratios^16^ (Mg/Si=∼1.0–1.1), whereas the olivine experiment (Mg/Si=∼1.8) simulates the alteration of a more ultramafic rocky core formed by large-scale silicate melting. An aqueous solution of NH_3_ and NaHCO_3_ was used for starting solution (see Methods). Using a steel-alloy autoclave (Supplementary Fig. 1), we simulate hydrothermal reactions within the rocky core of Enceladus by performing the experiments at pressure of 400 bar (∼150 km below the water–rock boundary). The possibility of the occurrence of hydrothermal reactions at the ocean–rock interface of Enceladus' ocean will be discussed below in the Discussion section. The experimental conditions are summarized in Supplementary Table 1.

### Dissolved gases and metals

**Figure 1:**
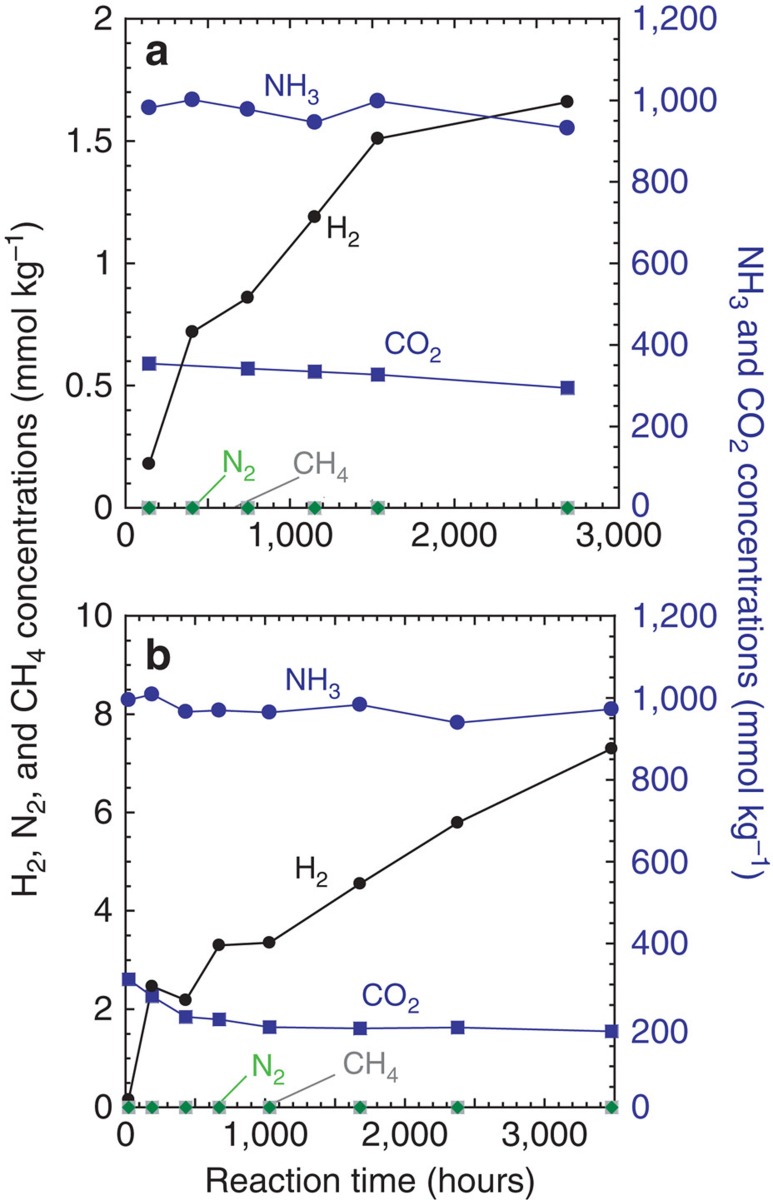
Variations in the concentrations of dissolved gas species. Results of H_2_, N_2_, CH_4_, ΣCO_2_ (=CO_2(aq)_+CO_3_^2−^+HCO_3_^−^) and ΣNH_3_ (=NH_3(aq)_+NH_4_^+^) during the experiments of (**a**) the opx experiment at 300 °C, and (**b**) the olivine experiment at 300 °C. Dissolved H_2_ was generated through the oxidation of Fe(II) in olivine to magnetite and serpentine, which were observed in the rocks after the experiments. The decreasing ΣCO_2_ is due to the formation of carbonate in the solid phase. The ΣNH_3_ concentrations are high and almost constant during the experiments. Our results provide no evidence for CH_4_ or N_2_ production from CO_2_ or NH_3_, respectively (CH_4_ production <5 μmol kg^−1^; N_2_ production <50 μmol kg^−1^) (see Supplementary Note 1). The experimental data are given in the Supplementary Table 2.

The measured concentrations of dissolved gas species and metallic ions in the fluid samples of the experiments can be compared directly with Cassini's observations of Enceladus' plume compositions^1^,^3^. Based on chemical equilibrium^17^,^18^, the lack of abundant N_2_ in the plumes^19^ might suggest the absence of hydrothermal activity, as it was proposed that N_2_ should form by the decomposition of NH_3_ at high temperatures^17^,^18^ (≥200 °C). However, our experimental results indicate that no N_2_ was produced from NH_3_ and that NH_3_ remains unaltered even at 300 °C (Fig. 1 and Supplementary Fig. 2) (N_2_ production <∼50 μmol kg^−1^ H_2_O: also see Supplementary Table 2 and Supplementary Note 1). These results indicate that the decomposition of NH_3_ is kinetically inhibited and is not catalysed by olivine, pyroxene or their alteration minerals under our experimental conditions. Given high activation energy for reducing-oxidizing reactions of N_2_, it has been suggested that catalysts would be required to promote these reactions at 500–1000 °C (refs 20, 21, 22). Typical catalysts attempted for decomposition of aqueous NH_3_ are platinum group, transition metals or their oxides^20^,^21^. However, these catalytic decomposition reactions of aqueous NH_3_ at high temperatures usually requires a significant amount of effective oxidants such as O_2_ (refs 20, 21, 22), which is probably unavailable in Enceladus. Thus, our experimental results indicate that the lack of N_2_ in the plumes^19^ is not indicative of the absence of hydrothermal reactions.

Our results also show that the conversion of CO_2_ to CH_4_ is suppressed (Fig. 1) (also see Supplementary Fig. 2, Supplementary Table 2, and Supplementary Note 1), as reported previously^23^,^24^. Based on chemical equilibrium calculations, previous studies hypothetically discuss the conversion of CO_2_ into CH_4_ under hydrothermal conditions in Enceladus^17^,^18^. In fact, given the presence of metallic grains, such as Fe-Ni alloy, in meteorites, the conversions of CO_2_ to CH_4_ would have proceeded in Enceladus through Fischer–Tropsch-type reactions^25^. However, McCollom and Seewald^23^ showed that these metallic catalysts were rapidly deactivated over time^23^, suggesting the loss of catalytic activity under sub-to-supercritical conditions over geological timescales. Thus, we suggest that the presence of abundance of CO_2_ in the plume^3^ also does not indicate the absence of hydrothermal reactions in Enceladus.

Furthermore, Fe^2+^, Mg^2+^ and Ca^2+^ become depleted in alkaline hydrothermal fluids (Supplementary Fig. 3 and Supplementary Table 3). These elements tend to be distributed in the rocky phase under alkaline hydrothermal conditions. Thus, the non-detection of these elements in the solid components of the plume^1^ also supports the proposition that the solution compositions of Enceladus' ocean are controlled mainly by hydrothermal reactions involving alteration minerals under alkaline conditions.

### Dissolved silica concentration

**Figure 2:**
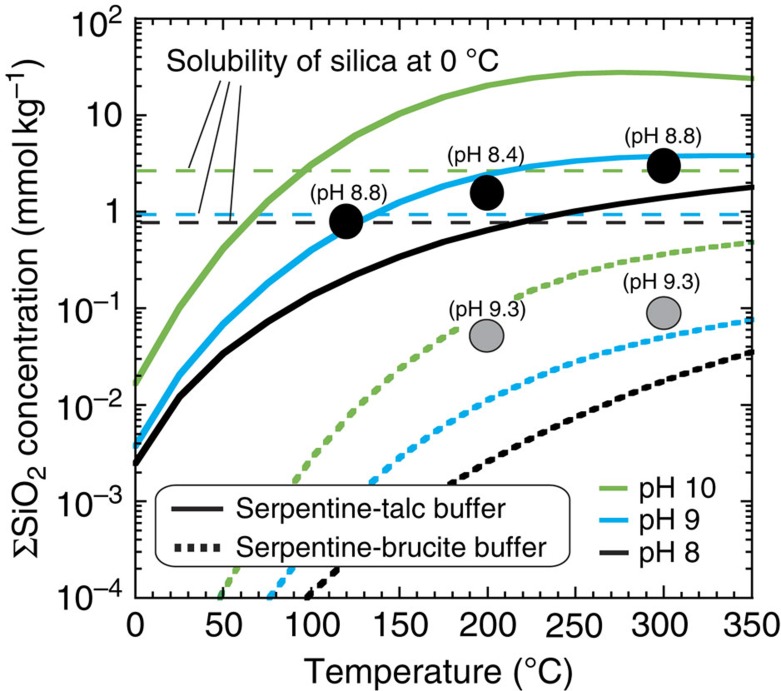
Experimental and calculation results of dissolved silica concentrations. Results of ΣSiO_2_ (=SiO_2(aq)_+HSiO_3_^−^+NaSiO_3(aq)_) are shown as a function of temperature at variable pH. Black and grey circles are the measured ΣSiO_2_ in fluid samples of the opx and olivine experiments, respectively. The results of total silica concentrations in the opx experiments are also given in ref. 5. The numbers annotated to the experimental data are the calculated in situ pH values (see Methods). Solid lines are ΣSiO_2_ values in chemical equilibrium at 400 bars according to the following reaction between serpentine and saponite/talc (serpentine–talc buffer): serpentine+2SiO_2(aq)_↔talc (saponite)+H_2_O. Dotted lines are ΣSiO_2_ values in chemical equilibrium at 400 bars according to the following reaction between serpentine and brucite (serpentine–brucite buffer): serpentine+H_2_O↔3brucite+2SiO_2(aq)_. The concentrations of HSiO_3_^−^ and NaHSiO_3(aq)_ were calculated for different pH values and at a constant Na^+^ concentration (100 mmol kg^−1^) using the equilibrium constants of the following reactions: SiO_2(aq)_+H_2_O↔HSiO_3_^−^+H^+^ and HSiO_3_^−^+Na^+^↔NaHSiO_3(aq)_. Horizontal broken lines show the solubility of amorphous silica at 0 °C and 100 bars for each pH value.

We observed considerable variability in the dissolved total silica concentrations (ΣSiO_2_=SiO_2(aq)_+HSiO_3_^−^+NaHSiO_3(aq)_) measured at the end of the experiments (Fig. 2). The presence of unaltered starting minerals in solid samples collected after the experiments (Supplementary Figs 4–6) and the observed continuous H_2_ formation during the experiments (Fig. 1) indicate that mineral alteration was still occurring after 3–10 months of the reaction time. However, ΣSiO_2_ in fluid samples reached steady levels within several months of reaction time (Supplementary Fig. 3). These steady-state levels of ΣSiO_2_ in the opx experiments are always much higher than those in the olivine experiments (Fig. 2). For instance, at 300 °C, ΣSiO_2_ in the opx experiment was ∼30 times that in the olivine experiment (Fig. 2). Fig. 2 also illustrates that ΣSiO_2_ generally increases with reaction temperature. In the opx experiments, ΣSiO_2_ at 300 °C was higher than that at 120 °C by a factor of ∼4.

To understand the factors that determine the observed trends, mineralogical and chemical analyses of the hydrothermally altered solid samples were performed (Supplementary Figs 4–6 and Supplementary Table 4). The major alteration products of the olivine experiments were serpentine (chrysotile), along with brucite, magnetite and carbonate (magnesite and dolomite), which is consistent with previous experiments and calculations of terrestrial ultramafic-based hydrothermal vents^26^,^27^ and with the proposed surface materials on Ceres^15^. In contrast, the alteration products of the opx experiments were dominated by serpentine (chrysotile) and saponite, along with talc, magnetite and carbonate (also see ref. 5), which are typical of carbonaceous chondrites^16^. Given the efficient oxidization of Fe(II) via high-temperature hydrothermal reactions^26^,^27^ (≥150 °C), the molar ratio of Mg to Si in the starting minerals is important in determining the major compositions of the alteration minerals. Serpentine and brucite are dominant in hydrous minerals when the Mg/Si ratio is high (Mg/Si>1.5), whereas serpentine and saponite/talc are the major hydrous silicates when the starting minerals are Si rich (Mg/Si<1.5).

In geothermal fields on Earth, the ΣSiO_2_ value of fluids is thought to be strongly influenced by reactions of alteration minerals, including serpentine, brucite and talc^28^. Fig. 2 shows the calculated equilibrium concentrations of ΣSiO_2_ for the reaction between serpentine and talc (that is, serpentine+2SiO_2(aq)_↔talc (saponite)+H_2_O: serpentine–talc buffer) and between serpentine and brucite (that is, serpentine+H_2_O↔3brucite+2SiO_2(aq)_: serpentine–brucite buffer) (also see the caption of Fig. 2). Fig. 2 indicates that the calculated values of ΣSiO_2_ for the two buffer systems are in good agreement with the measured values of ΣSiO_2_ in the opx and olivine experiments, respectively. Because of the relatively slow formation of the alteration minerals in the experiments, the measured ΣSiO_2_ would not be controlled by the buffer systems for the initial periods of reaction time (Supplementary Fig. 3). However, as the alteration reactions proceed, the measured ΣSiO_2_ contents are determined by the chemical equilibrium of the buffer systems, regardless of the presence of unaltered starting minerals. These results strongly suggest that the ΣSiO_2_ content of fluids in Enceladus' interior is also controlled by the buffer systems of the alteration minerals over geological timescales.

The formation of nanosilica colloids in a cooling silica-saturated solution explains Cassini's observations^5^. In this scenario, high-temperature fluids in chemical equilibrium with rocks of the core enter and mix with a low-temperature ocean^5^. Colloidal silica nanoparticles form upon cooling in the ocean when the ΣSiO_2_ content of hydrothermal fluids exceeded the solubility of amorphous silica^5^. As in a previous associated study^5^, the present study assumes that silica nanoparticles are generated in an ocean at 0 °C (ref. 4), which provides a lower limit on the required temperature of hydrothermal reactions. Fig. 2 shows that ΣSiO_2_ for the serpentine–talc buffer exceeds the solubility of silica at 0 °C when the fluid temperature becomes sufficiently high^5^ (≥90 °C). In contrast, ΣSiO_2_ for the serpentine–brucite buffer is much lower than the solubility of silica for fluid temperatures of ≤350 °C. These results suggest that to sustain high ΣSiO_2_ contents sufficient to form silica nanoparticles, hydrous silicates on Enceladus should have been dominated by serpentine and saponite/talc, which are similar phases to those found in carbonaceous chondrites^16^. These results further imply that Enceladus' rocky core would not have experienced large-scale silicate melting and formation of more ultramafic rocks, if hydrothermal reactions took place in the core.

## Discussion

**Figure 3:**
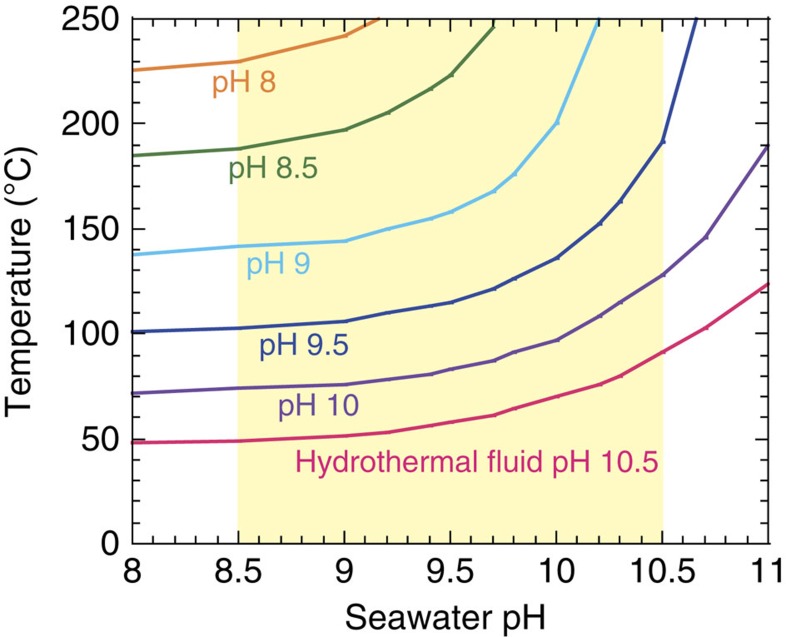
Required minimum temperatures of hydrothermal fluids on Enceladus. Results are obtained from the cross-sections between the solubility of silica at 0 °C and the ΣSiO_2_ values determined by the serpentine–talc buffer as a function of seawater pH at 0 °C for different values of hydrothermal fluid pH. In other words, the required minimum temperatures are determined when the ΣSiO_2_ value for a given hydrothermal fluid exceeds the solubility of silica at 0 °C. The silica solubility is obtained for Na^+^ concentration of 0.1 mol kg^−1^ and pressure 30 bar. The pH of Enceladus' seawater has been suggested to be in the range of 8.5–10.5 (refs 1, 5) (the shaded area). The solid lines show the results when hydrothermal fluid pH values change to seawater pH values by cooling and mixing with oceanic water (see text).

A previous study indicates that to form silica nanoparticles, the temperature of fluids on Enceladus needs to exceed ∼90 °C if fluid pH remains constant upon cooling^5^. However, fluid pH is highly likely to change upon cooling and mixing with seawater. Fig. 3 shows the minimum temperatures of hydrothermal reactions for the serpentine–talc buffer for different fluid pH values as a function of seawater pH, required to produce silica nanoparticles on Enceladus. Enceladus' seawater is suggested to be mildly alkaline (pH∼8.5–10.5), based on both the composition of emitted salt-rich grains^1^,^2^ and the stable existence of silica nanoparticles^5^. On the other hand, pH values of pore water in the rocky core are only roughly constrained^5^ (pH>∼8.5).

If the ocean-core system in Enceladus is chemically closed to other volatile reservoirs, such as the icy crust, the pH values of hydrothermal fluids and the ocean would be controlled by water–rock interactions^11^. In such a chemically closed system, fluid pH tends to increase upon cooling^11^. In fact, our experimental results show that the pH values of fluids range 8–9 at high temperatures (120–300 °C) and increase to ∼10 upon cooling to the room temperature (∼15 °C) in the opx experiments (Supplementary Table 3). This is because the dissociation constant of H_2_O to H^+^ and OH^−^ has a maximum at 200–300 °C, and because the conversion of NH_3_ and H^+^ to NH_4_^+^ tends to proceed at lower temperatures. Our experimental results show that the thermal decomposition of NH_3_ to N_2_ is efficiently inhibited even at high temperatures (Fig. 1), which, in turn, facilitates the increases in fluid pH upon cooling. These results suggest that pH values of NH_3_-containing fluids increase, possibly by one unit or more, upon cooling. If pH values increase by one unit upon cooling, required temperatures become ∼200 °C for seawater pH of 8.5–10.5 (Fig. 3), as shown previously^5^. Given that the required temperatures may vary within ∼20 °C depending on Na^+^ and ΣCO_2_ concentrations and pressure^5^, hydrothermal activity at ≥∼150–200 °C is required to account for the formation of silica nanoparticles on Enceladus for a chemically closed, ocean-core system in Enceladus.

On the other hand, if Enceladus' ocean is chemically open to the icy crust through effective volatile exchanges^10^, pH values of fluids and ocean are not determined simply by a change in dissolved species upon cooling. In fact, pH values of hydrothermal fluids may possibly be close to, or even higher than, those of oceanic water if there is a significant difference in ΣCO_2_ concentrations in solutions between the ocean and hydrothermal fluids. Such differences in ΣCO_2_ can occur when pore water contains a lower ΣCO_2_ concentration than the seawater due to formation of carbonates and organic matter by interactions with the rocks; whereas a high ΣCO_2_ concentration in the ocean is sustained by a supply from CO_2_ clathrates in the icy crust^10^. In this case, pH values of the pore water could be moderately to strongly alkaline because of low abundances of CO_3_^2−^ and HCO_3_^−^, and those of the ocean would be mildly alkaline buffered by a NaHCO_3_ or NaCO_3_ system^1^,^29^. Thus, the existence of a ΣCO_2_ gradient in Enceladus would lower the minimum temperatures of water–rock interactions required for the formation of silica nanoparticles, compared with those for a closed system (Fig. 3). However, strongly alkaline solutions (pH=11–13) buffered by a NaOH system^29^ are unlikely to occur within Enceladus because of the presence of abundance of CO_2_ in the interior^3^. In addition, given a plausible rapid water circulation in Enceladus inferred from the size of silica nanoparticles expelled by the plumes^5^, a large difference in ΣCO_2_ between the ocean and core tends to be mitigated over geological timescales. Thus, even when Enceladus' ocean is largely affected by CO_2_ supply from the icy crust, we conclude that hydrothermal fluids should be moderately alkaline at most (pH∼8.5–10.5). In the extreme case of fluid pH of 10.5 and seawater pH of 8.5 within the range constrained by the previous studies^1^,^5^, the minimum temperature required to form silica nanoparticles becomes ∼50 °C (Fig. 3). A lower pH value of hydrothermal fluids requires a higher minimum temperature for the formation of silica nanoparticles (Fig. 3). Thus, we conclude that whether Enceladus' ocean is chemically open or closed, high-temperature water–rock reactions (>∼50 °C) would be required in the interior.

It has been indicated that the silica nanoparticles of 2–8 nm in radius observed in Saturn's stream particles must have been formed by recent or ongoing hydrothermal activity on Enceladus, because the growth of several nm sized silica particles, for example, by Ostwald ripening, in the subsurface ocean would take months to several years at most^5^. Our experimental results provide additional supporting evidence for the presence of ongoing hydrothermal activity on Enceladus. We show that chemical equilibrium between dissolved silica and alteration minerals is achieved within months, even at a relatively low hydrothermal temperature of 120 °C (Supplementary Fig. 3). These results suggest that if the interior of Enceladus had become completely cold (<<100 °C) and hydrothermal reactions had ceased, then ΣSiO_2_ in the ocean would have reached low levels over a geologically short time, as determined by the equilibrium at low temperatures (Fig. 2).

We estimated the dissolution rate of silica nanoparticles in an unsaturated solution. Previous work shows that the dissolution rate of silica nanoparticles in pure water at 0 °C and pH 5.7 is 8.8 × 10^−15^ cm s^−1^ (∼3 nm year^−1^)^30^. Under these conditions, even a several nm sized particle is estimated to dissolve in a few years. In an alkaline and NaCl-rich solution, the dissolution proceeds much more quickly (by one or two orders of magnitude) than that in pure water^30^,^31^. This suggests that silica nanoparticles would have readily dissolved over geological timescales after ΣSiO_2_ in the ocean fell below the solubility of silica. Accordingly, the formation of silica nanoparticles is most likely sustained by geologically recent or ongoing hydrothermal activity.

**Figure 4:**
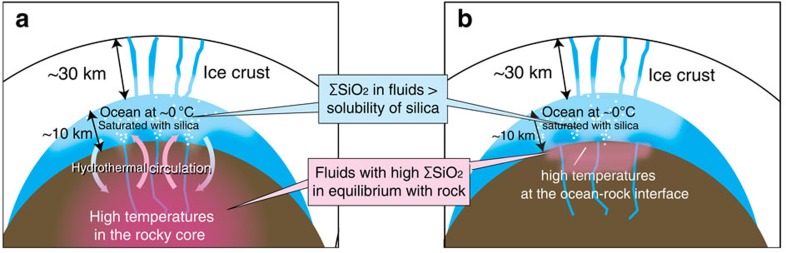
Schematic illustration of hydrothermal circulations and the formation of silica on Enceladus. (**a**) Deep hydrothermal circulation would have occurred between a warm, and probably porous^5^,^6^,^7^,^45^,^70^, rocky core and a cold ocean because of remnant heat from the early stages of Enceladus' evolution. (**b**) If heating has been induced by a recent heating event such as crustal overturn^41^, orbital evolution^42^ or an impact^43^, hydrothermal reactions would have taken place at the ocean–rock interface. In this case, serpentinization and its associated heat production may have been important in sustaining high-temperature water–rock interactions.

Although the energy budget of Enceladus' current geological activity remains unclear^32^,^33^, constraints derived from the observations of silica nanoparticles may help our understanding of the interior structure and thermal evolution of Enceladus. We show that the rocky core of Enceladus is most likely composed of Si-rich, carbonaceous chondritic rocks and that the core has not experienced large-scale silicate melting and therefore remains porous. This view is in agreement with the recent findings of a low-density core as inferred from the gravity data^6^,^7^. These results regarding the properties of the rocky core imply that the oceanic water could penetrate deep below the ocean–rock interface^34^, resulting in deep hydrothermal circulation driven by remnant heat of the early stages of Enceladus' evolution stored in the deep core (Fig. 4a). Thermal evolution models suggest that Enceladus' core reached high temperatures due to short-lived radiogenic heating and was dehydrated in the early stage of its evolution^35^,^36^, if it formed within 4 million years (Myrs) of the formation of the solar system. Such radiogenic heat together with steady and episodic tidal dissipation heating could be retained in the deep core for a long time (on the order of 2 billion years (Gyrs) or more)^37^. In this case, exothermic re-serpentinization of the deep core would have subsequently occurred and could have kept the interior warm for longer^37^. A formation age of Enceladus, and thus the Saturnian system, within 4 Myrs of the formation age of the solar system is consistent with the formation age proposed for Iapetus^38^ and with the typical lifetime of protoplanetary disks around the Sun-like stars^39^,^40^.

However, given that a porous rocky core tends to lose remnant heat rapidly, especially if it is percolated by the oceanic water, it may be more likely that hydrothermal activity on Enceladus was triggered by a recent incidental heating event (for example, a catastrophic crustal overturn^41^, an orbital evolution^42^ or an impact^43^). The thickness of plume particles deposits on the small Saturnian satellites also implies that the duration of cryovolcanic activities on Enceladus would be as short as 10 Myrs (ref. 44). These incidental heating event could have increased the temperature near the ocean–rock interface (Fig. 4b). It is highly uncertain whether this event alone could have produced a sufficient amount of heat to cause hydrothermal activity, because such an event provides heat mainly in the icy shell rather than in the rocks of the seafloor. However, if Enceladus' rocky core is fragmented, the incidental events would have triggered effective tidal dissipation within the core^45^, especially near the ocean–rock interface. In addition, if the ocean–rock interface had contained pristine minerals, such an event might have initiated ice melting and subsequent exothermic serpentinization. This in turn could have triggered a positive feedback between serpentinization, temperature increase and large tidal dissipation^32^,^45^, possibly leading to hydrothermal reactions. Numerical simulations of Europa's ocean^46^,^47^ show that hydrothermal plumes produce upwelling currents at a velocity of 1–5 cm s^−1^. Although simulations for Enceladus' ocean are required to evaluate the intensity of upwelling currents, these results imply that hydrothermal plumes^46^,^47^ and water convection^8^,^48^ in the ocean could have transported nanoparticles from the seafloor to the plume source near the ice–ocean interface.

We propose that the temperature of ongoing hydrothermal reactions on Enceladus may be sufficiently high to cause effective Fe(II) oxidization associated with serpentinization, especially when the reactions occur in a relatively chemically closed system. However, if Enceladus has been warm since its formation, the rocky core might have already become completely serpentinized and oxidized by past water–rock reactions^34^. The available data on Enceladus' plumes are insufficient to determine whether hydrothermal activity is involved in ongoing serpentinization and Fe(II) oxidization. Our experimental results (Fig. 1 and Supplementary Fig. 2) and theoretical modelling^12^ indicate that further evidence for ongoing serpentinization on Enceladus would be high levels of H_2_ in the plumes (>>1 mmol kg^−1^), which may be testable via *in situ* measurements by Cassini and future missions. Although Cassini's observations during a recent series of low-velocity flybys of Enceladus show that the presence of substantial abundances of H_2_ in the plume^10^, it is still unclear whether the hydrogen is native or generated by reactions with titanium wall of the INMS antechamber^3^. Serpentinization could also support the emergence and survival of possible chemoautotrophic life on Enceladus through the provision of reducing power (that is, H_2_) into CO_2_-rich water, as proposed for early Earth and Mars^49^,^50^. Our experiments suggest that H_2_ generation on Enceladus is as efficient as that in terrestrial ultramafic-hosted hydrothermal vents^27^ (Fig. 2), where H_2_-based microbial ecosystems are sustained^51^.

## Methods

### Hydrothermal experiments

The apparatus and methodology of the hydrothermal experiments conducted in the present study were based on the previous study simulating terrestrial hydrothermal vents^52^,^53^. Supplementary Fig. 1 shows the schematic diagram of the flexible gold reaction cell and steel (Inconel)-alloy autoclave used in the present study. The flexible cell consisted of a gold bag with a titanium head (Supplementary Fig. 1). The surface of the titanium head was oxidized by combustion before use to avoid catalytic reactions on the surface of metallic titanium. The inside wall of the sampling tube was coated with gold to avoid catalytic reactions. The flexible gold reaction cell was heated at 500 °C for 3 h in air to remove potential contamination of organic matter before each experiment.

Olivine used in the experiments of the present study was extracted from mantle peridotite originated from San Carlos (Mg_1.8_Fe_0.2_SiO_4_). As natural olivine was used, it included minor minerals such as orthopyroxene, clinopyroxene and spinel, which have provided Al, Ca, and other elements in the fluids and alteration minerals (see the caption of Supplementary Fig. 6). In addition, trace amounts of transition elements, such as Ni and Mn, were also contained in olivine. Orthoenstatie crystals were synthesized by the flux method^54^. Special grade reagents of MgO and SiO_2_ were mixed with enstatite stoichiometry, and added to the flux of special grade Li_2_O_3_, MoO_3_, and V_2_O_5_ that was mixed in the proportions of 34.3, 55.9 and 9.8 wt.%, respectively. The weight ratio of the nutriment (that is, MgO+SiO_2_) to the flux was 0.05. The mixture of nutriment and flux were heated in a platinum crucible at 970 °C for 100 h in the air, and then cooled to 730 °C at an average cooling rate of 2 °C h^−1^ and to room temperature at ∼100 °C h^−1^. Synthesized orthoenstatite crystals were separated from the solvent by washing in hot water. In the opx experiments, we mixed the synthesised opx with San Carlos olivine (the bulk composition of the opx-olivine mixtures: Mg/Si=∼1.2) to reproduce the Mg/Si ratios of carbonaceous chondrites (Mg/Si=∼1.0–1.1). Given the stoichiometry of alteration reactions, alteration mineral assemblages would change drastically at Mg/Si=∼1.5 (see the text); accordingly, we consider that the rock mixtures of the opx experiments can approximately simulate the reactions of carbonaceous chondritic rocks.

The initial concentrations of NH_3_ and NaHCO_3_ in the starting solution were 1.1 × 10^3^ and 3.6 × 10^2^ mmol kg^−1^ H_2_O, or 2% and 0.7% relative to H_2_O, respectively. These concentrations are comparable to volatile compositions of comets^55^ (NH_3_: ∼1% relative to H_2_O) and to Na^+^ abundances observed in Enceladus' plumes^1^ (∼1–3 × 10^2^ mmol kg^−1^ H_2_O), respectively. The ΣCO_2_ (=CO_2(aq)_+CO_3_^2−^+HCO_3_^−^) concentrations in the experiments may be 1/2 to 1/10 of the CO_2_ abundance in comets^55^. However, the plume activities might have resulted in a loss of CO_2_ throughout the history of Enceladus^8^, and the change in ΣCO_2_ would not change our conclusions significantly. Isotopic labelling was used for the species in the starting aqueous solution (that is, ^15^NH_3_ with 1% of ^15^N; NaH^13^CO_3_ with 10% of ^13^C) to verify the products of hydrothermal reactions, such as CH_4_ and N_2_.

The starting minerals were powdered with an alumina mortar and ultrasonically cleaned with acetone first and then pure water, before use. The size of the powdered minerals was typically ∼10–200 μm. Mixtures of aqueous solution (∼60 g) and starting minerals (∼15 g) were introduced into the reaction cell. The initial water/rock ratio was fixed at ∼4 in the experiments, because the water/rock ratio in submarine hydrothermal environments on Earth is considered to be limited to <∼5 (ref. 56). During the experiments, fluid samples of ∼2 g were collected. Thus, the water/rock ratio decreased to ∼3 at the end of the experiments because of fluid sampling.

During the experiments, the flexible cell collapses as fluids are removed during sampling, which allowed us to conduct online sampling of the fluids at a near constant temperature and pressure condition without vapour phase present^52^,^53^. Glass and Teflon vials purged with pure Ar gas were connected with the sampling valve and used to sample fluids in the flexible cell (Supplementary Fig. 1). The blank experiments (that is, without starting minerals or dissolved species) were performed in our previous study^57^, which used the same experimental system as the present study. The H_2_ concentration throughout the blank experiment is ∼0.008 mmol kg^−1^ or less^57^, which is negligible compared with the H_2_ concentrations in our experiments (Fig. 1). More detailed descriptions on the experimental systems may be found in refs 52, 57.

The experiments were conducted at a constant pressure of 400 bar, corresponding to the pressure of the interior of Enceladus' core. The temperature conditions of the olivine experiments were 200 and 300 °C, and those of the opx experiments were 120, 200 and 300 °C (Supplementary Table 1). In the opx experiments at 120 and 200 °C, we started the experiment at 120 °C for the first ∼2 months, and then increased the temperature to 200 °C and continued the experiment for another ∼1 month. The other experiments were performed at constant temperatures. The durations of the experiments were 2–10 months.

Chemical analyses of dissolved gas species were performed using a gas chromatograph (GC-2014 Shimadzu) and ion chromatograph (ICS-1600 DIONEX) at JAMSTEC, Japan Agency for Marine-Earth Science and Technology, and a gas chromatograph-mass spectrometer (GCMS-QP2010 Shimazdu) at the University of Tokyo. Inductively coupled plasma atomic emission spectroscopy (PerkinElmer) was also conducted at JAMSTEC to measure the concentrations of dissolved elements. Mineralogical and chemical analyses of the rocks were conducted using an X-ray diffraction spectrometer (X'PERT-PRO PANanlytical) and a scanning electron microscope with an electron probe microanalyser (JXA-8200 JEOL) at the University of Tokyo.

### Equilibrium calculations

For calculating ΣSiO_2_ determined by the equilibrium of secondary minerals and the solubility of amorphous silica at 0 °C, we used the equilibrium constants computed by the SUPCRT92 program^58^. Although Na-rich saponite was the major alteration mineral found in samples collected after the opx experiments, we used thermodynamic data for talc [Mg_3_Si_4_O_10_(OH)_2_] rather than for Na-rich saponite [(Na)_0.3_Mg_3_(Si,Al)_4_O_10_(OH)_2_]. This is because of the absence of Na-rich saponite in the database^58^ and the similarity in the chemical formulae of these minerals. We also used the thermodynamic data of amorphous silica for nanoparticles. This assumption provides a lower limit of the temperature required for hydrothermal reactions to form nanosilica, because silica nanoparticles are less stable than amorphous silica because of the difference in surface free energy.

### *In situ* pH calculations

*In situ* pH (pH_*in situ*_) was calculated with the Geochemist's Workbench computer code^59^ based on pH at room temperature (pH_25_°_C_) and concentrations of dissolved elements and species in fluids collected at the final sampling of the experiments (Supplementary Tables 2 and 3). In this pH_*in situ*_ calculations, charge balance was constrained from the pH_25_°_C_ value, while Na was used as the element compensating the imbalanced charge derived from analytical errors. The thermodynamic database required for this calculation was generated by the SUPCRT92 computer program^58^, with thermodynamic data for mineral, aqueous species and complexes from refs 60, 61, 62, 63, 64, 65. The B-dot activity model was used^66^,^67^. The temperature-dependent activity coefficient for aqueous CO_2_ was derived from the empirical relationship of ref. 68 and the temperature-dependent activity of water in a NaCl solution was derived from the formulation of ref. 59. Cleverley and Bastrakov^69^ provide useful temperature-dependent polynomial functions for both these last two parameters^69^. Although the calculations were carried out using a total pressure of 500 bars, pressure is a minor factor since the equilibrium constants are not sensitive to the modest changes in pressure.

## Additional information

**How to cite this article:** Sekine, Y. *et al.* High-temperature water–rock interactions and hydrothermal environments in the chondrite-like core of Enceladus. *Nat. Commun.* 6:8604 doi: 10.1038/ncomms9604 (2015).

## Supplementary Material

Supplementary Information Supplementary Figures 1-6, Supplementary Tables 1-4, Supplementary Note 1 and Supplementary References

## References

[b1] Postberg F. *et al.* Sodium salts in E-ring ice grains from an ocean below the surface of Enceladus. Nature 459, 1098–1101 (2009). 19553992 10.1038/nature08046

[b2] Postberg F., Schmidt J., Hillier J., Kempf S. & Srama R. A salt-water reservoir as the source of a compositionally stratified plume on Enceladus. Nature 474, 620–622 (2011). 21697830 10.1038/nature10175

[b3] Waite J. H. Jr *et al.* Liquid water on Enceladus from observations of ammonia and ^40^Ar in the plume. Nature 460, 487–490 (2009).

[b4] Schmidt J., Brilliantov N., Spahn F. & Kempf S. Slow dust in Enceladus' plume from condensation and wall collisions in tiger stripe fractures. Nature 451, 685–688 (2008). 18256665 10.1038/nature06491

[b5] Hsu H.-W. *et al.* Silica nanoparticles as an evidence of hydrothermal activities at Enceladus. Nature 519, 207–210 (2015). 25762281 10.1038/nature14262

[b6] Iess L. *et al.* The gravity field and interior structure of Enceladus. Science 344, 78–80 (2014). 24700854 10.1126/science.1250551

[b7] McKinnon W. B. Effect of Enceladus's rapid synchronous spin on interpretation of Cassini gravity. Geophys. Res. Lett. 42, 2137–2143 (2015).

[b8] Matson D. L., Castillo-Rogez J. C., Davies A. G. & Johnson T. V. Enceladus: a hypothesis for bringing both heat and chemicals to the surface. Icarus 221, 53–62 (2012).

[b9] Mousis O. *et al.* Formation conditions of Enceladus and origin of its methane reservoir. Astrophys. J. Lett. 701, 39–42 (2009).

[b10] Bouquet A., Mousis O., Waite J. H. & Picaud S. Possible evidence for a methane source in Enceladus' ocean. Geophys. Res. Lett. 42, 1334–1339 (2015).

[b11] Zolotov M. Y. An oceanic composition on early and today's Enceladus. Geophys. Res. Lett. 34, L23203 (2007).

[b12] Glein C. R., Baross J. A. & Waite J. H. Jr The pH of Enceladus' ocean. Geochim. Cosmochim. Acta 162, 202–219 (2015).

[b13] Nakamura T. *et al.* Chondrulelike objects in short-period comet 81 P/Wild 2. Science 321, 1664–1667 (2008). 18801994 10.1126/science.1160995

[b14] Crovisier J. *et al.* The spectrum of comet Hale-Bopp (C/1995 O1) observed with the infrared space observatory at 2.9 astronomical units from the sun. Science 275, 1904–1907 (1997). 9072960 10.1126/science.275.5308.1904

[b15] Milliken R. E. & Rivkin A. S. Brucite and carbonate assemblages from altered olivine-rich materials on Ceres. Nat. Geosci. 2, 258–261 (2009).

[b16] Brearley A. J. in Meteorites and the Early Solar System II (eds Lauretta D. S., McSween H. Y. 587–624University of Arizona Press (2006).

[b17] Glein C. R., Zolotov M. Y. & Shock E. L. The oxidation state of hydrothermal systems on early Enceladus. Icarus 197, 157–163 (2008).

[b18] Matson D. L., Castillo J. C., Lunine J. & Johnson T. V. Enceladus' plume: compositional evidence for a hot interior. Icarus 187, 569–573 (2007).

[b19] Hansen C. J. *et al.* The composition and structure of the Enceladus plume. Geophys. Res. Lett. 38, L11202 (2011).

[b20] Lee D. K. Mechanism and kinetics of the catalytic oxidation of aqueous ammonia to molecular nitrogen. Environ. Sci. Technol. 37, 5745–5749 (2003). 14717189 10.1021/es034332q

[b21] Oshima Y., Inaba K. & Koda S. Catalytic supercritical water oxidation of coke works waste with manganese oxide. Sekiyu Gakkaishi 44, 343–350 (2001).

[b22] Helling R. K. & Tester J. W. Oxidation of simple compounds and mixtures in supercritical water: carbon monoxide, ammonia, and ethanol. Envion. Sci. Technol. 22, 1319–1324 (1988).

[b23] McCollom T. M. & Seewald J. S. Experimental constraints on the hydrothermal reactivity of organic acids and acid anions: I. Formic acid and formate. Geochim. Cosmochim. Acta 67, 3625–3644 (2003).

[b24] McCollom T. M., Lollar B. S., Lacrampe-Couloume G. & Seewald J. S. The influence of carbon source on abiotic organic synthesis and carbon isotope fractionation under hydrothermal conditions. Geochim. Cosmochim. Acta 74, 2717–2740 (2010).

[b25] Horita J. & Berndt M. E. Abiogenic methane formation and isotopic fractionation under hydrothermal conditions. Science 285, 1055–1057 (1999). 10446049 10.1126/science.285.5430.1055

[b26] Seyfried W. E. Jr, Foustoukos D. I. & Fu Q. Redox evolution and mass transfer during serpentinization: an experimental and theoretical study at 200 °C, 500 bar with implications for ultramafic-hosted hydrothermal systems at mid-ocean ridges. Geochim. Cosmochim. Acta 71, 3872–3886 (2007).

[b27] McCollom T. M. & Bach W. Thermodynamic constraints on hydrogen generation during serpentinization of ultramafic rocks. Geochim. Cosmochim. Acta 73, 856–875 (2009).

[b28] Frost B. R. & Beard J. S. On silica activity and serpentinization. J. Petrol. 48, 1351–1368 (2007).

[b29] Zolotov M. Y. Aqueous fluid composition in CI chondritic materials: chemical equilibrium assessments in closed systems. Icarus 220, 713–729 (2012).

[b30] Icenhower J. P. & Dove P. M. The dissolution kinetics of amorphous silica into sodium chloride solutions: effects of temperature and ionic strength. Geochim. Cosmochim. Acta 64, 4193–4203 (2000).

[b31] Brantley S. L. in Kinetics of Water-Rock Interaction (eds Kubicki J. D., White A. F. 151–210Springer (2008).

[b32] Spencer J. R. *et al.* in Saturn from Cassini-Huygens (eds Dougherty M. K., Esposito L. W., Krimigis S. M. 683–724Springer (2005).

[b33] Shoji D., Hussmann H., Sohl F. & Kurita K. Non-steady state tidal heating of Enceladus. Icarus 235, 75–85 (2014).

[b34] Vance S. *et al.* Hydrothermal systems in small ocean planets. Astrobiology 7, 987–1005 (2007). 18163874 10.1089/ast.2007.0075

[b35] Castillo-Rogez J. C., Matson D. L., Vance S. D., Davies A. G. & Johnson T. V. in *Proceedings of the 38th Lunar and Planetary* *Science* Vol. 38, 2265 (League City, 2007).

[b36] Schubert G., Anderson J. D., Travis B. J. & Palguta J. Enceladus: present internal structure and differentiation by early and long-term radiogenic heating. Icarus 188, 345–355 (2007).

[b37] Travis B. J. & Schubert G. Keeping Enceladus warm. Icarus 250, 32–42 (2015).

[b38] Castillo-Rogez J. *et al.* ^26^Al-decay: heat production and a revised age for Iapetus. Icarus 204, 658–662 (2009).

[b39] Wyatt M. C. Evolution of debris disks. Annu. Rev. Astron. Astrophys. 46, 339–383 (2008).

[b40] Pascucci I. & Tachibana S. in Protoplanetary Dust: Astrophysical and Cosmochemical Perspectives (eds Apai D., Lauretta D. S. 263–298Cambridge University Press (2010).

[b41] O'Neill C. O. & Nimmo F. The role of episodic overturn in generating the surface geology and heat flow on Enceladus. Nat. Geosci 3, 88–91 (2010).

[b42] Wisdom J. Spin-orbit secondary resonance dynamics of Enceladus. Astron. J. 128, 484–491 (2004).

[b43] Roberts J. H. & Stickle A. M. *Proceedings of the 46th Lunar* *and Planetary Science* Vol. 46, 1468 (The Woodlands, 2015).

[b44] Hirata N., Miyamoto H. & Showman A. P. Particle deposition on the Saturnian satellites from ephemeral cryovolcanism on Enceladus. Geophys. Res. Lett. 41, 4135–4141 (2014).

[b45] Roberts J. H. The fluffy core of Enceladus. Icarus 258, 54–66 (2015).

[b46] Vance S. & Goodman J. in Europa (eds Pappalardo R. T., McKinnon W. B., Khurana K. 459–482University of Arizona Press (2006).

[b47] Goodman J. C. & Lenferink E. Numerical simulations of marine hydrothermal plumes for Europa and other icy worlds. Icarus 221, 970–983 (2012).

[b48] Soderlund K. M., Schmidt B. E., Wicht J. & Blankenship D. D. Ocean-driven heating of Europa's icy shell at low latitudes. Nat. Geosci. 7, 16–19 (2014).

[b49] Russell M. J., Hall A. J. & Martin W. Serpentinization as a source of energy at the origin of life. Geobiology 8, 355–371 (2010). 20572872 10.1111/j.1472-4669.2010.00249.x

[b50] Schulte M., Blake D., Hoehler T. & McCollom T. Serpentinization and its implications for life on the early Earth and Mars. Astrobiology 6, 364–376 (2006). 16689652 10.1089/ast.2006.6.364

[b51] Kelley D. S. *et al.* A serpentine-hosted ecosystem: the Lost City hydrothermal field. Science 307, 1428–1434 (2005). 15746419 10.1126/science.1102556

[b52] Shibuya T. *et al.* Reactions between basalt and CO_2_-rich seawater at 250 and 350 °C, 500 bars: Implications for the CO_2_ sequestration into the modern oceanic crust and composition of hydrothermal vent fluid in the CO_2_-rich early ocean. Chem. Geol. 359, 1–9 (2013).

[b53] Seyfried W. E. Jr Experimental and theoretical constraints on hydrothermal alteration processes at Mid-Ocean Ridges. Annu. Rev. Earth Planet. Sci. 15, 317–335 (1987).

[b54] Tachibana S., Tsuchiyama A. & Nagahara H. Experimental study of incongruent evaporation kinetics of enstatite in vacuum and in hydrogen gas. Geochim. Cosmochim. Acta 66, 713–728 (2002).

[b55] Bocklelée-Morvan D., Crovisier J., Mumma M. J. & Weaver H. A. in Comets II eds Festou M. C., Keller H. U., Weaver H. A. 391–423University Arizona Press (2004).

[b56] Wetzel L. R. & Shock E. L. Distinguishing ultramafic—from basalt-hosted submarine hydrothermal systems by comparing calculated vent fluid compositions. J. Geophys. Res. 105, 8319–8340 (2000).

[b57] Yoshizaki M. *et al.* H_2_ generation by experimental hydrothermal alteration of komatiitic glass at 300 °C and 500 bars: A preliminary result from on-going experiment. Geochem. J. 43, 17–22 (2009).

[b58] Johnson J. W., Oelkers E. H. & Helgeson H. C. SUPCRT92: a software package for calculating the standard molal thermodynamic properties of minerals, gases, aqueous species, and reactions from 1 to 5000 bar and 0 to 1000 °C. Comput. Geosci. 18, 899–947 (1992).

[b59] Bethke C. M. Geochemical and Biogeochemical Reaction Modeling Cambridge University Press (2008).

[b60] Shock E. L. & Helgeson H. C. Calculation of the thermodynamic and transport properties of aqueous species at high pressures and temperatures: correlation algorithms for ionic species and equation of state predictions to 5 kb and 1000 °C. Geochim. Cosmochim. Acta. 52, 2009–2036 (1988).

[b61] Shock E. L. & Koretsky C. M. Metal-organic complexes in geochemical processes: Estimation of standard partial molal thermodynamic properties of aqueous complexes between metal cations and monovalent organic acid ligands at high pressures and temperatures. Geochim. Cosmochim. Acta. 59, 1497–1532 (1995).

[b62] Shock E. L., Helgeson H. C. & Sverjensky D. A. Calculation of the thermodynamic and transport properties of aqueous species at high pressures and temperatures: standard partial molal properties of inorganic neutral species. Geochim. Cosmochim. Acta. 53, 2157–2183 (1989).

[b63] Shock E. L., Sassani D. C., Willis M. & Sverjensky D. A. Inorganic species in geologic fluids: correlations among standard molal thermodynamic properties of aqueous ions and hydroxide complexes. Geochim. Cosmochim. Acta. 61, 907–950 (1997). 11541225 10.1016/s0016-7037(96)00339-0

[b64] Sverjensky D. A., Shock E. L. & Helgeson H. C. Prediction of the thermodynamic properties of aqueous metal complexes to 1000 °C and 5 kb. Geochim. Cosmochim. Acta. 61, 1359–1412 (1997). 11541435 10.1016/s0016-7037(97)00009-4

[b65] McCollom T. M. & Shock E. L. Geochemical constraints on chemolithoautotrophic metabolism by microorganisms in seafloor hydrothermal systems. Geochim. Cosmochim. Acta. 61, 4375–4391 (1997). 11541662 10.1016/s0016-7037(97)00241-x

[b66] Helgeson H. C. Thermodynamics of hydrothermal systems at elevated temperatures and pressures. Am. J. Sci. 267, 729–804 (1969).

[b67] Helgeson H. C. & Kirkham D. H. Theoretical prediction of the thermodynamic behavior of aquerous electrolytes at high pressures and temperatures: II. Debye-Huckel parameters for activity coefficients and relative partial molal properties. Am. J. Sci. 1199–1261 (1974).

[b68] Drummond S. E. Boiling and Mixing of Hydrothermal Fluids: Chemical Effects on Mineral Precipitation PhD thesis Pennsylvania State University (1981).

[b69] Cleverley J. S. & Bastrakov E. N. K2GWB: utility for generating thermodynamic data files for the Geochemist's Workbench ® at 0–1000 °C and 1–5000 bar from UT2K and the UNITHERM database. Comput. Geosci. 31, 756–767 (2005).

[b70] Collins G. C. & Goodman J. C. Enceladus' south polar sea. Icarus 189, 72–82 (2007).

