# Supplementary material for: High-temperature water–rock interactions and hydrothermal environments in the chondrite-like core of Enceladus
Source: Nat Commun. 2015 Oct 27;6:8604. doi: 10.1038/ncomms9604 (PMC4639802; doi:10.1038/ncomms9604)
Supplement: Supplementary Information — Supplementary Figures 1-6, Supplementary Tables 1-4, Supplementary Note 1 and Supplementary References [file ncomms9604-s1.pdf]

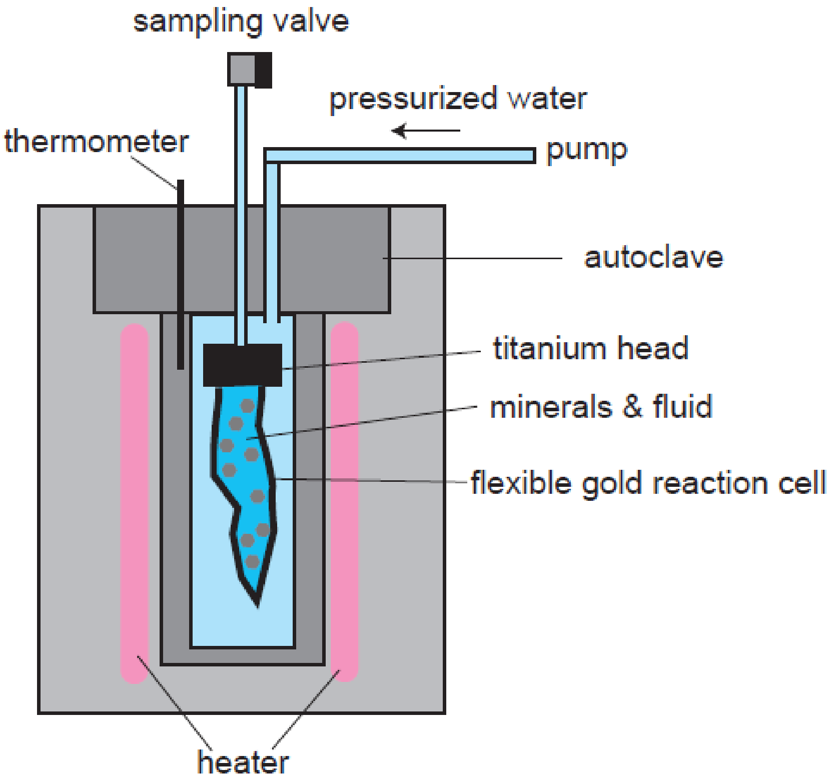

Supplementary Fig. 1. Schematic diagram of the steel-alloy autoclave. Minerals and fluids are contained in a flexible gold reaction cell, which is pressurized and heated in the autoclave.

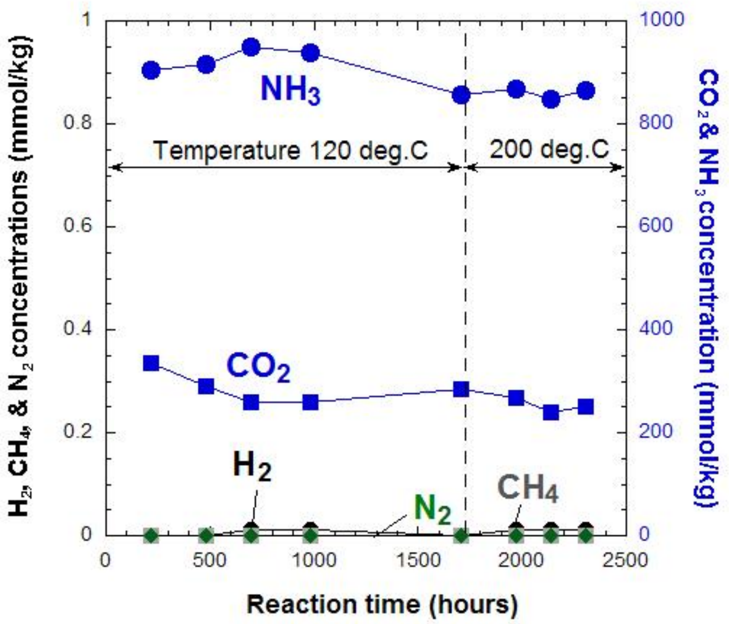

(a)

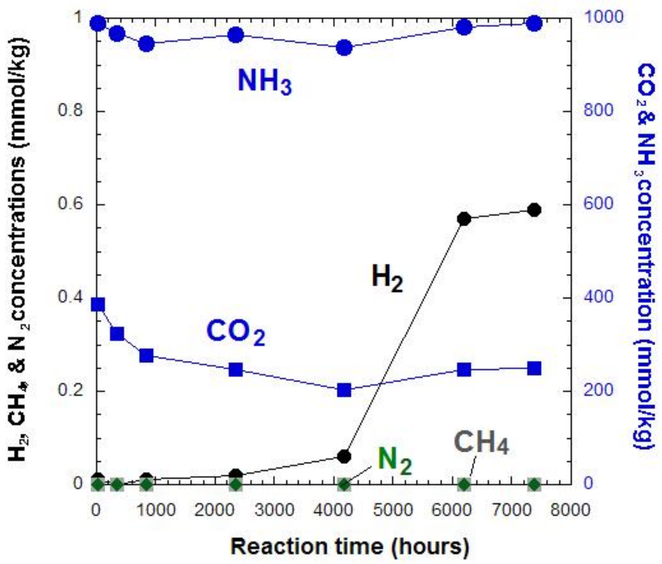

(b)

Supplementary Fig. 2. The time variations in dissolved gas species. Results are shown for a) the opx experiment at 120 and 200°C, and b) the olivine experiment at 200°C.

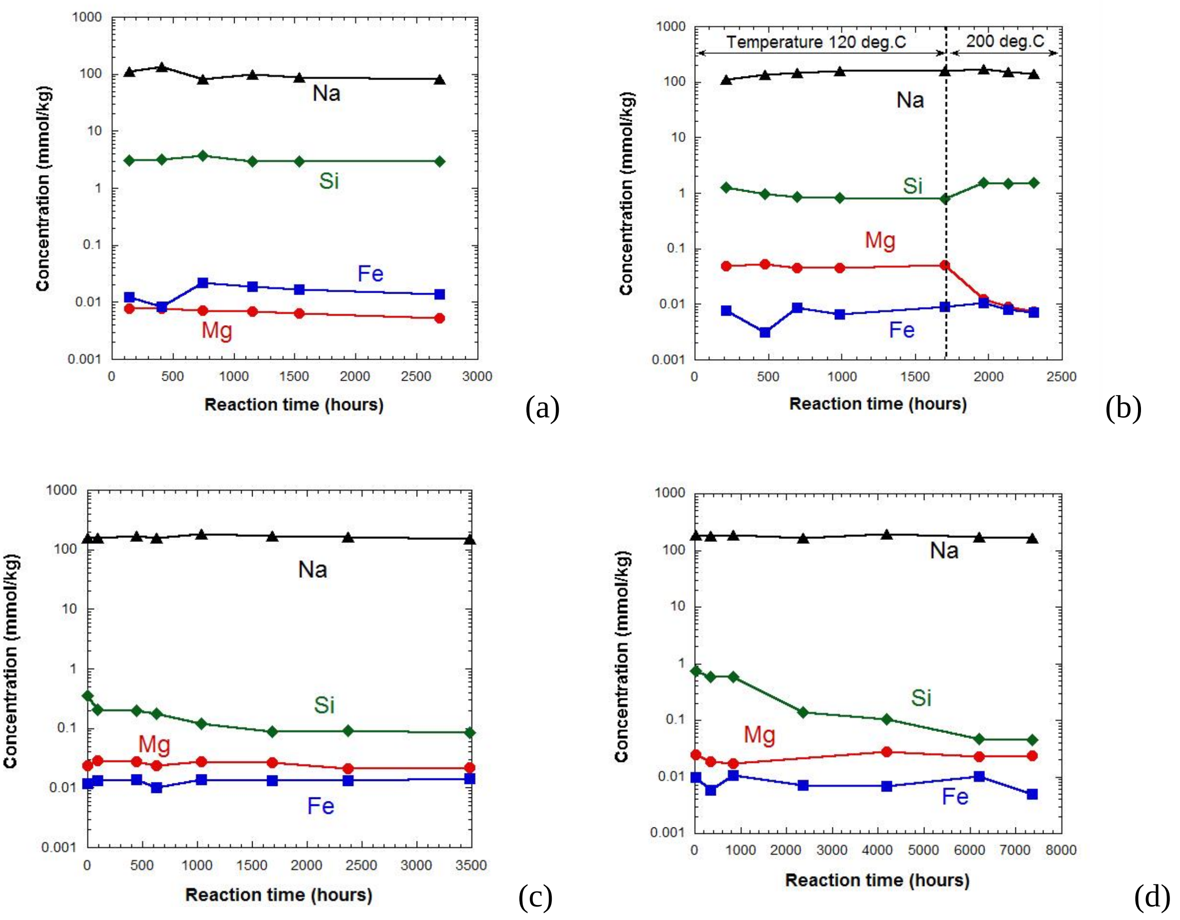

Supplementary Fig. 3. The time variations in concentration of selected elements. Results of Na, Si, Mg, and Fe are shown for a) the opx experiment at 300°C, b) the opx experiment at 120 and 200°C, c) olivine experiment at 300°C, and d) the olivine experiment at 200°C.

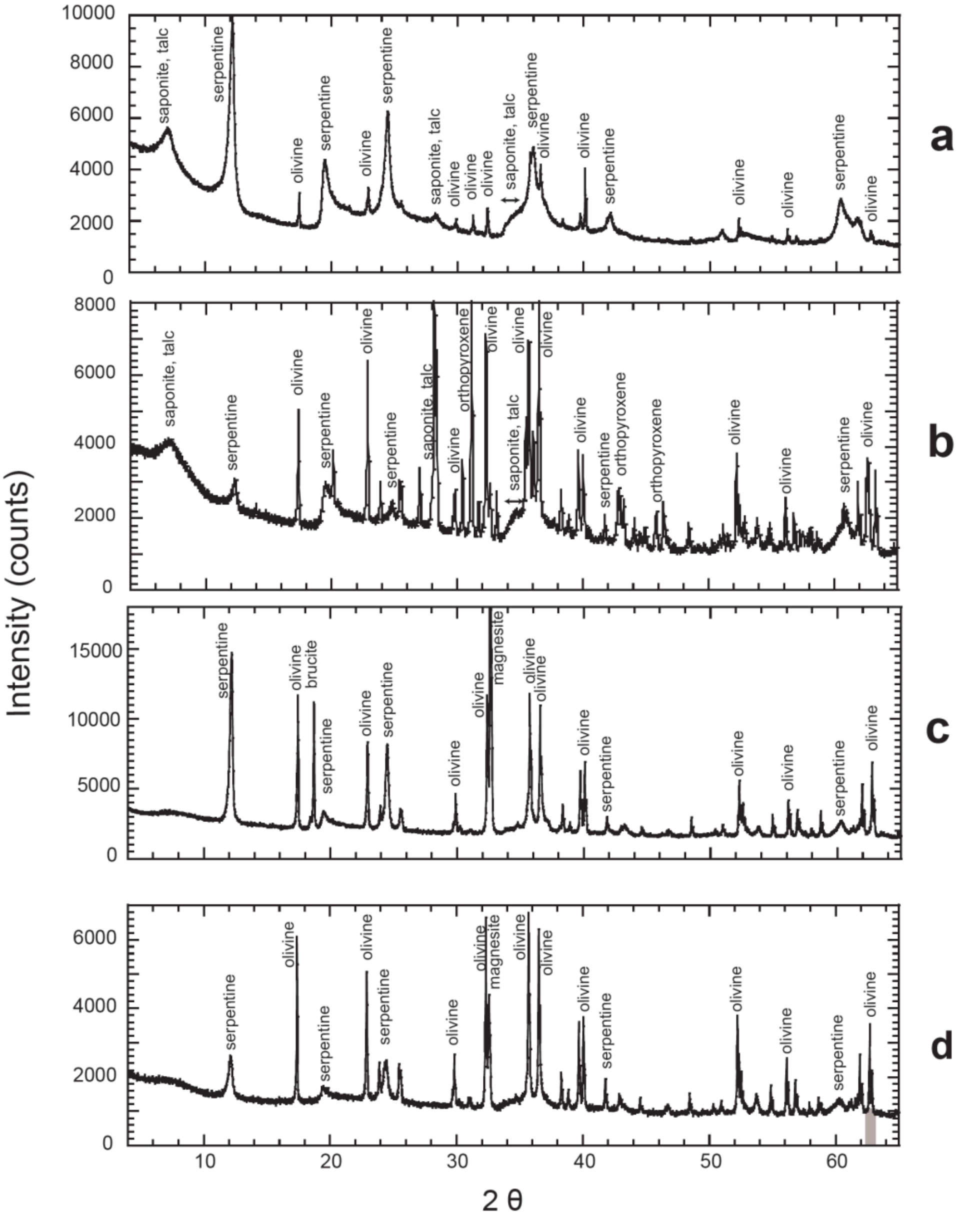

Supplementary Fig. 4. X-ray diffraction (XRD) spectra of the mineral residues. Results are shown for a) the opx experiment at 300°C, b) the opx experiment at 120 and 200°C, c) the olivine experiment at 300°C, and d) the olivine experiment at 200°C. Minerals correspond to the XRD peaks are also shown. For the opx experiments, serpentine and saponite are the major alteration minerals. For the olivine experiments at 300°C, there are distinct XRD signals attributed from serpentine, brucite, and magnesite with faint peaks of magnetite. At 200°C, the XRD signals of brucite and magnetite become very small compared with those at 300°C, which is consistent with the results of electron probe microanalyzer (EPMA) analyses shown below. In the lower-temperature experiments, the strong peaks of the starting minerals (i.e., olivine and orthopyroxene) are observed both in the opx and olivine experiments.

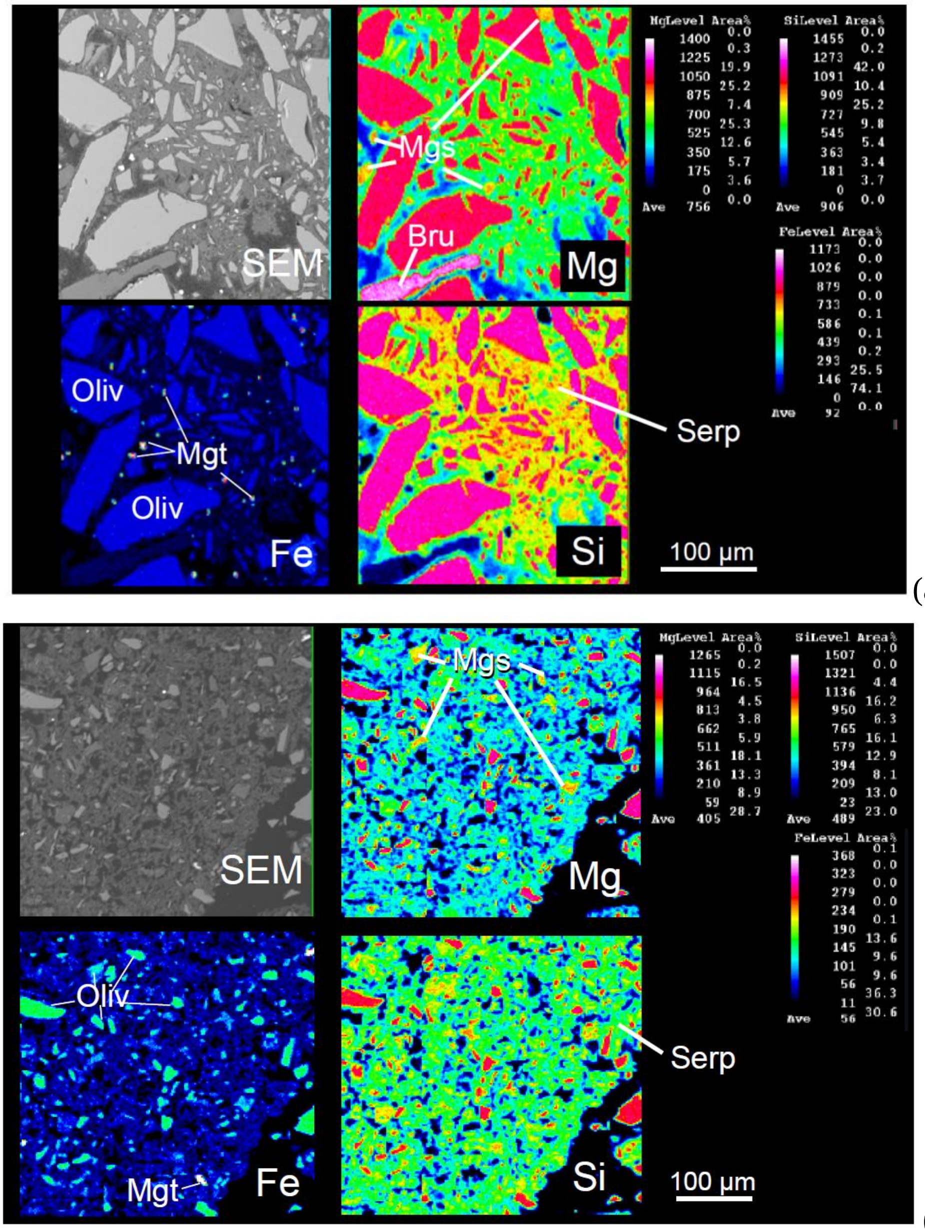

(a) (b)

Supplementary Fig. 5. Typical SEM images and elemental mapping for the rock residues collected after the olivine experiments. Results are shown for the experiments at (a) 300°C and (b) 200°C. Oliv: olivine, Serp: serpentine, Mgt: magnetite, Bru: brucite, and Mgs: magnesite. This figure and Supplementary Table 4 indicate the formation of serpentine, magnetite, brucite, and Mg-rich carbonate in the olivine experiments at 300°C. On the other hand, brucite and magnetite are less abundant in the rock residues at 200°C, which is consistent with the previous experiment of olivine serpentinization conducted at 200°C (ref. 1).

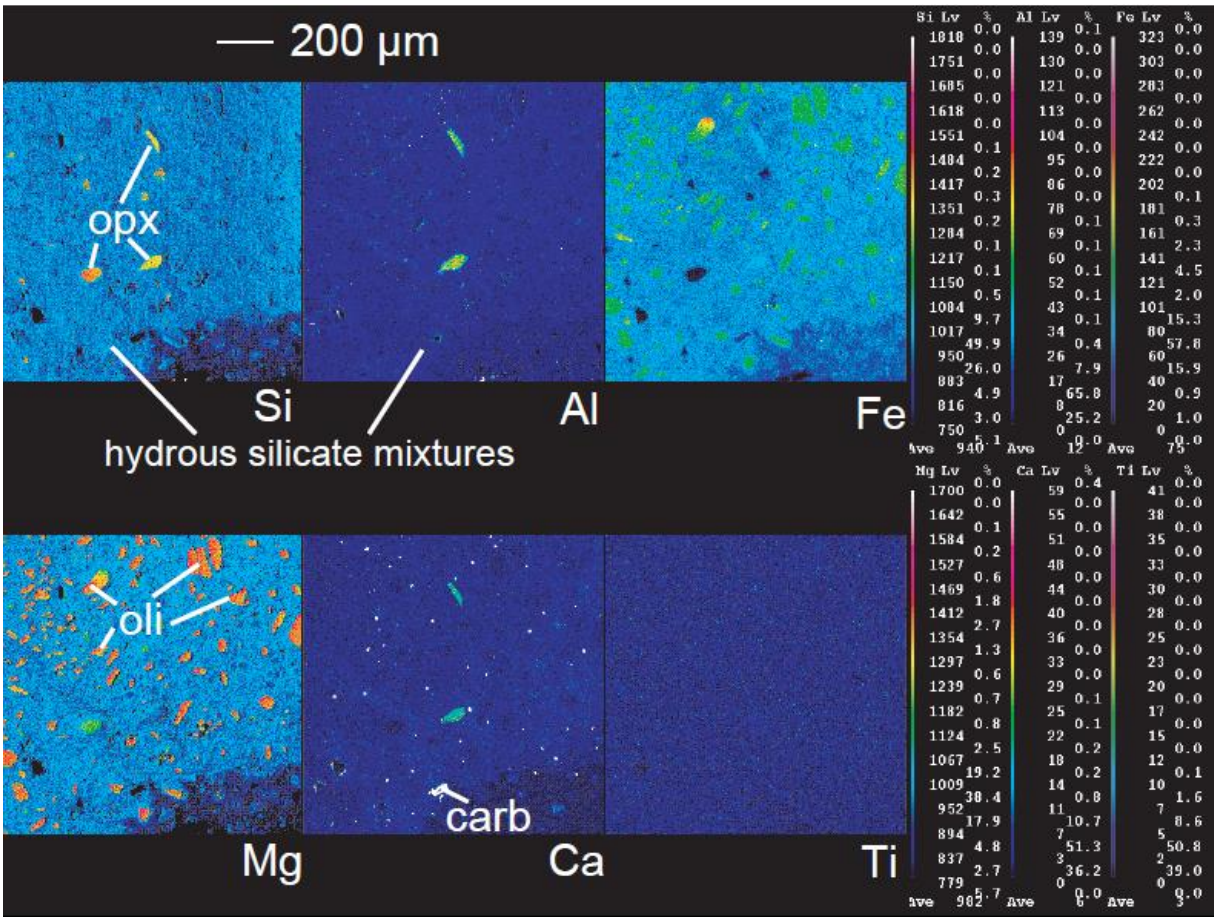

Supplementary Fig. 6. Elemental mapping of the rock residues collected after the opx experiment at 300°C. Oliv: olivine, Opx: orthopyroxene. Hydrous silicate mixture in the figure would be composed of serpentine, saponite, and talc given their chemical compositions (Supplementary Table 4) and XRD data (Supplementary Fig. 4). We found some unreacted olivine and opx mineral grains in the rock residue. In contrast to the olivine experiments, serpentine, saponite, and talc coexist in the matrix of the rock residue, although there are variations in Mg, Si, and Al contents in the coexistent mixtures (Supplementary Table 4). Some of the unreacted opx crystals contain ~2–3 wt.% of Al_2O_3 (we term these "Al-bearing opx", and a typical chemical composition is shown in Supplementary Table 4). Given the similarity of this chemical composition to that of opx crystals retrieved from San Carlos peridotite[2], these Al-bearing opx crystals would have been included in starting minerals as a minor mineral phase of San Carlos olivine. Spinel inclusions in San Carlos olivine would also be included in the starting minerals[3].These Al-bearing opx crystals and spinel inclusions are considered to be two of the sources of aluminium in the saponite-rich matrix.

Supplementary Table 1. A summary of experimental conditions and major alteration minerals

starting minerals	temperature	pressure	initial NH_3	initial $NaHCO_3$	major alteration minerals
orthopyroxene + olivine	300°C	400 bar	1.1 mol/kg	360 mmol/kg	saponite, serpentine
orthopyroxene + olivine	120°C (0-1704 hrs of reaction time)<br><br>200°C (1704-2304 hrs of reaction time)	400 bar	1.1 mol/kg	360 mmol/kg	saponite serpentine
olivine	300°C	400 bar	1.1 mol/kg	360 mmol/kg	serpentine, brucite, magnesite, magnetite
olivine	200°C	400 bar	1.1 mol/kg	360 mmol/kg	serpentine, brucite, magnesite, magnetite

Supplementary Table 2. Time variations in dissolved volatiles in fluids (in mmol/kg H_2O).

Opx experiment at 300℃

time (hours)	ΣCO_2	H_2	NH_3	CH_4	N_2
144	354	0.18	982	<0.005	<0.05
408	n/a	0.72	1002	<0.005	<0.05
744	342	0.86	978	<0.005	<0.05
1152	335	1.19	946	<0.005	<0.05
1536	327	1.51	998	<0.005	<0.05
2688	294	1.66	932	<0.005	<0.05

Opx experiment at 120 and 200℃

time (hours)	ΣCO_2	H_2	NH_3	CH_4	N_2
**initial temperature = 120℃**					
216	336	0.00	905	<0.005	<0.05
480	289	0.00	916	<0.005	<0.05
696	259	0.01	950	<0.005	<0.05
984	260	0.01	937	<0.005	<0.05
1704	284	0.00	856	<0.005	<0.05
**temperature increased to 200℃**					
1968	268	0.01	869	<0.005	<0.05
2136	239	0.01	848	<0.005	<0.05
2304	252	0.01	866	<0.005	<0.05

Supplementary Table 2. (Continued)

Olivine experiment at 300℃

time (hours)	ΣCO_2	H_2	NH_3	CH_4	N_2
24	313	0.16	995	<0.005	<0.05
192	271	2.46	1008	<0.005	<0.05
432	221	2.18	966	<0.005	<0.05
672	215	3.30	969	<0.005	<0.05
1032	196	3.35	964	<0.005	<0.05
1680	193	4.55	983	<0.005	<0.05
2376	195	5.79	939	<0.005	<0.05
3480	186	7.30	972	<0.005	<0.05

Olivine experiment at 200℃

time (hours)	ΣCO_2	H_2	NH_3	CH_4	N_2
24	387	0.01	988	<0.005	<0.05
336	324	0.00	966	<0.005	<0.05
840	277	0.01	946	<0.005	<0.05
2352	247	0.02	965	<0.005	<0.05
4176	203	0.06	936	<0.005	<0.05
6192	246	0.57	982	<0.005	<0.05
7368	249	0.59	988	<0.005	<0.05

Supplementary Table 3. The time variations in dissolved major elements (in mmol/kg H_2O) and pH at room temperature of fluid samples. In-situ pH is calculated with the methodology described in the Methods section.

Opx experiment at 300℃

time (hours)	Mg	Fe	Si	Na	Ca	K	Al	pH at room T
144	0.00762	0.0124	3.06	109	0.0180	0.256	0.0213	10
408	0.00770	0.0083	3.21	134	0.0095	0.203	0.0190	10.5
744	0.00708	0.0220	3.79	82	0.0066	0.171	0.0229	10
1152	0.00678	0.0186	2.91	98	0.0162	0.166	0.0230	10.6
1536	0.00636	0.0167	2.91	89	0.0098	0.164	0.0263	10.5
2688	0.00525	0.0137	2.90	82	0.0071	0.160	0.0252	10.1
								in-situ pH 8.8

Opx experiment at 120 and 200℃

time (hours)	Mg	Fe	Si	Na	Ca	K	Al	pH at room T
**initial temperature = 120℃**								
216	0.04811	0.0077	1.25	113	0.0293	0.202	0.0293	10.1
480	0.05240	0.0031	0.98	136	0.0246	0.207	0.0378	9.8
696	0.04477	0.0088	0.84	146	0.0238	0.205	0.0184	10.2
984	0.04490	0.0065	0.81	158	0.0206	0.198	0.0196	10
1704	0.05012	0.0089	0.78	159	0.0133	0.201	0.0212	10.2
**temperature increased to 200℃**								in-situ pH 8.8
1968	0.01227	0.0107	1.55	168	0.0101	0.187	0.0398	10
2136	0.00887	0.0080	1.51	152	0.0110	0.173	0.0340	10
2304	0.00743	0.0072	1.55	141	0.0114	0.167	0.0279	10.2
								in-situ pH 8.4

Olivine experiment at 300℃

time (hours)	Mg	Fe	Si	Na	Ca	K	Al	pH at room T
24	0.0240	0.0117	0.355	157	0.0123	0.418	0.0307	10.5
192	0.0286	0.0131	0.208	156	0.0132	0.415	0.0270	10.1
432	0.0280	0.0140	0.197	167	0.0116	0.416	0.0223	9.8
672	0.0233	0.0102	0.173	158	0.0141	0.426	0.0189	9.9
1032	0.0275	0.0136	0.119	183	0.0163	0.424	0.0186	9.9
1680	0.0264	0.0135	0.088	169	0.0117	0.417	0.0214	11.2
2376	0.0213	0.0135	0.090	166	0.0075	0.425	0.0200	10
3480	0.0220	0.0143	0.085	151	0.0111	0.409	0.0182	10.5
								in-situ pH 9.3

Supplementary Table 3. (Continued)

Olivine experiment at 200℃

time (hours)	Mg	Fe	Si	Na	Ca	K	Al	pH at room T
24	0.0247	0.0097	0.749	188	0.0063	0.409	0.0062	10.2
336	0.0188	0.0057	0.581	177	0.0067	0.405	0.0069	10.2
840	0.0169	0.0105	0.595	185	0.0045	0.415	0.0070	10.6
2352	0.0249	0.0072	0.137	168	0.0031	0.395	0.0076	10.2
4176	0.0283	0.0068	0.106	194	0.0038	0.404	0.0105	10.5
6192	0.0224	0.0101	0.048	171	0.0027	0.424	0.0087	10.5
7368	0.0235	0.0050	0.046	168	0.0031	0.363	0.0105	10.6
								in-situ pH 9.3

Supplementary Table 4. Results of electron probe microanalyzer (EPMA) analyses for typical alteration and unreacted minerals contained in the rock residues formed in the olivine and opx experiments at 300°C. The values are in weight percent. These alteration minerals frequently coexist in the rock residues. Thus, the results may not be explained by a simple chemical composition of alteration mineral.

	SiO_2	TiO_2	Al_2O_3	FeO	MnO	MgO	CaO	Na_2O	K_2O	Total
**Olivine exp.**										
Serpentine	32.73	0.09	0.33	1.78	0.00	31.90	0.03	0.14	0.01	67.01
Brucite (with serpentine and $Fe(OH)_2$)	3.64	0.04	0.56	5.10	0.18	72.97	0.03	0.01	0.01	82.54
Olivine	40.17	0.03	0.00	9.29	0.13	51.09	0.09	0.00	0.00	100.80
**Opx exp.**										
Saponite-rich matrix	37.87	0.03	1.11	5.25	0.09	26.81	0.10	0.05	0.00	71.30
Serpentine-rich matrix	34.92	0.01	0.07	4.21	0.06	21.26	0.20	0.01	0.01	60.75
Olivine	39.84	0.00	0.05	9.59	0.17	49.96	0.07	0.00	0.00	99.68
Opx	58.82	0.01	0.00	0.13	0.00	40.93	0.03	0.03	0.02	99.97
Al-bearing opx	55.21	0.00	2.75	5.38	0.14	34.84	0.91	0.03	0.04	99.30

Supplementary Note 1

The time variations in dissolved elements and gas species ($\sum CO_2$, H_2, ΣNH_3, Mg, Fe, Si (=ΣSiO_2), Na, K, Ca, and Al in mmol/kg H_2O), pH at room temperature, and calculated in-situ pH at the termination of the experiments are summarized in Supplementary Table 2 and 3.

The variations in dissolved gas species in the opx and olivine experiments are shown in Fig. 1 and Supplementary Fig. 2. Compared with the results of the olivine experiment at 300°C (Fig. 1), H_2 concentration was low and increased abruptly and intermittently in the olivine experiment at 200°C (Supplementary Fig. 2b), as reported in the previous experiments[3]. According to the previous study[3], this is caused by less production of magnetite at 200°C, which is consistent with the mineralogical analyses of the rock residues by the present study (see Supplementary Fig. 4). At 200°C, serpentine contained relatively high contents of ferric and ferrous Fe as reported previously[3].

In the present study, the levels of CH_4 in fluid samples were at nearly constant throughout the experiments, ~0.01 mmol/kg. However, the proportion of ^{13}C in CH_4 in fluid samples remained within the range of the natural isotopic ratio, indicative of no evidence of CH_4 formation from dissolved CO_2 (CH_4 production <~5 μmol/kg). The CH_4 dissolved in the fluids were probably originated from dissociation of contaminated organic matter under high-temperature and high-pressure conditions.

The results of gas analyses showed that gaseous N_2 with ~0.1 mmol/kg were contained in the sampling vials; nonetheless, the proportion of ^{15}N in N_2 were also very low (within the range of the natural isotopic ratio), indicating that the N_2 in the fluid samples was probably air contamination during sampling. The presence of O_2 in the samples supports the idea of air contamination during sampling. These results indicate no production of N_2 from NH_3 (N_2 production <~50 μmol/kg) in our experiments. This conclusion is supported by the NH_3 concentrations in the solution. Our experimental results show that the NH_3 concentrations are almost constant (~900–1000 mmol/kg H_2O) during the experiments (Supplementary Fig. 2 and Table 2). Small decreases in NH_3 concentrations from the initial values may be caused by the incorporation of NH_4^+ in a smectite, such as saponite.

Although we cannot conclude whether catalytic reactions proceed in Enceladus at this stage, we consider that catalytic reactions would not have been sufficiently efficient to convert all of primordial CO_2 into CH_4 in Enceladus. More laboratory experiments using metallic alloys are required in the future to draw more realistic view of chemical evolution of primordial volatiles in Enceladus and other icy moons.

Supplementary Fig. 3 shows the variations in major elements in the fluid samples for the opx and olivine experiments. These results indicate that the abundances of dissolved element reached steady states within several months of reaction time. The reason for the gradual decreases in ΣSiO_2 in the experiment would be caused by a change in the buffer system in association with the slow formation of alteration minerals, such as serpentine, brucite, and saponite/talc at lower temperatures.

Supplementary references

1. Seyfried, Jr., W. E., Foustoukos, D. I., & Fu Q. Redox evolution and mass transfer during serpentinization: An experimental and theoretical study at 200°C, 500 bar with implications for ultramafic-hosted hydrothermal systems at mid-ocean ridges. *Geochim. Cosmochim. Acta* **71**, 3872–3886 (2007).

2. Xu, Y. & Shankland, T. J. Electrical conductivity of orthopyroxene and its high pressure phases. *Geophys. Res. Lett*. **26**, 2645–2648 (1999).

3. Mayhew, L. E., Ellison, E. T., McCollom, T. M., Trainor, T. P., & Templeton, A. S. Hydrogen generation from low-temperature water–rock reactions. *Nat. Geosci.* **6**, 478–484 (2013).
